# Haploinsufficiency of Activation-Induced Deaminase for Antibody Diversification and Chromosome Translocations both *In Vitro* and *In Vivo*

**DOI:** 10.1371/journal.pone.0003927

**Published:** 2008-12-12

**Authors:** Isora V. Sernández, Virginia G. de Yébenes, Yair Dorsett, Almudena R. Ramiro

**Affiliations:** 1 DNA Hypermutation and Cancer Group, Spanish National Cancer Research Center (CNIO), Madrid, Spain; 2 The Rockefeller University, New York, New York, United States of America; Albert Einstein College of Medicine, United States of America

## Abstract

The humoral immune response critically relies on the secondary diversification of antibodies. This diversification takes places through somatic remodelling of the antibody genes by two molecular mechanisms, Class Switch Recombination (CSR) and Somatic Hypermutation (SHM). The enzyme Activation Induced Cytidine Deaminase (AID) initiates both SHM and CSR by deaminating cytosine residues on the DNA of immunoglobulin genes. While crucial for immunity, AID-catalysed deamination is also the triggering event for the generation of lymphomagenic chromosome translocations. To address whether restricting the levels of AID expression *in vivo* contributes to the regulation of its function, we analysed mice harbouring a single copy of the AID gene (AID^+/−^). AID^+/−^ mice express roughly 50% of normal AID levels, and display a mild hyperplasia, reminiscent of AID deficient mice and humans. Moreover, we found that AID^+/−^ cells have an impaired competence for CSR and SHM, which indicates that AID gene dose is limiting for its physiologic function. We next evaluated the impact of AID reduction in AID^+/−^ mice on the generation of chromosome translocations. Our results show that the frequency of AID-promoted c-myc/IgH translocations is reduced in AID^+/−^ mice, both *in vivo* and *in vitro*. Therefore, AID is haploinsufficient for antibody diversification and chromosome translocations. These findings suggest that limiting the physiologic levels of AID expression can be a regulatory mechanism that ensures an optimal balance between immune proficiency and genome integrity.

## Introduction

B cells are responsible for generating a repertoire of antibodies of virtually unlimited diversity in order to confront the antigenic universe. Antibody diversification is achieved through somatic remodelling of immunoglobulin (Ig) genes at two different stages of B cell differentiation. The first one is antigen- independent and takes place during B cell generation in the bone marrow through a site-specific recombination named V(D)J recombination, which gives rise to B cells expressing a primary repertoire of Iow affinity IgM antibodies (reviewed in [1]). Upon antigen encounter B cells have as yet another chance to further diversify their antibody repertoire in germinal centers by two independent molecular mechanisms called somatic hypermutation (SHM) and class switch recombination (CSR). SHM reshapes the antigen binding site of Igs by introducing nucleotide changes in their variable genes. B cells where SHM gives rise to antibodies with higher affinity for their cognate antigen are positively selected, a process referred to as affinity maturation (reviewed in [2] and [3]). CSR is a region-specific recombination reaction that replaces the primary μ constant (Cμ) region by a downstream constant region (Cγ, Cε or Cα), thereby generating antibodies endowed with new functions for pathogen neutralization while retaining the same antigen specificity. CSR takes place between highly repetitive sequences that precede the Cμ, Cγ, Cε and Cα genes, called switch regions, through the generation of double strand breaks (DSBs), ligation, and concomitant excision of the intervening sequence from the locus (reviewed in [4], [5]).

Both SHM and CSR are initiated by the very same enzyme, Activation Induced Cytidine Deaminase (AID) [6]. In humans, mutations in the AID gene are associated with a rare (1/2000000) immunodeficiency called Hyper IgM Syndrome type 2 (HIGM2) [7]. HIGM2 patients display impaired CSR and SHM and are prone to bacterial infections of the respiratory and digestive tracts [7]. AID initiates SHM and CSR by deaminating cytosine residues of the variable and switch regions of the Ig genes, respectively [8], [9], [10], [11], [12], [13]. Cytosine deamination on DNA converts a normal C:G pair into a U:G mismatch. AID-generated U:G mismatches are processed through uracil removal by Uracil-N-Glycosylase (UNG) or through recognition by the mismatch repair (MMR) machinery, which results in the generation of either a mutation (SHM) or a DSB (CSR) [11], [14], [15], [16].

Most of the lymphomas diagnosed in the western world arise from mature B cells and are characterized by the presence of chromosomal translocations that involve one of the Ig loci and a proto-oncogene [17], [18]. These translocations are known to play a role in the etiology of these B cell neoplasias [17], [18]. *In vivo* and *in vitro* studies have shown that AID can promote the generation of pro-lymphomagenic translocations [19], [20], [21], [22], and that CSR and the translocation reaction are initiated by a common pathway that involves DNA deamination and UNG [20]. The impact of AID function in B cell neoplasia development has been addressed in a number of *in vivo* models, including IL6 [19], [21] and pristane [23] promoted plasmacytomas, BCL-6-induced diffuse large B cell lymphoma [22], Eμ-Myc model of B cell lymphoma [24] and a myc-induced multiple myeloma model [25]. In all the cases absence of AID either delayed the onset or shifted the nature of the neoplasia towards a more immature origin, hence reinforcing the idea that AID expression plays a role in the generation of mature B cell lymphomas by promoting DNA lesions.

Therefore, AID function, while crucial to the development of an efficient immune response, can pose a risk to DNA stability in B cells. Different regulatory mechanisms may be responsible to minimize unwanted DNA damage by AID.

First, AID mutagenic activity is mostly limited to the Ig loci (reviewed in [26]), and although AID-induced lesions in other genes have been reported [27], [28], these events are rare. Second, AID accessibility to DNA is restrained by fine control of subcellular localization [29], [30]. Third, the presence of AID mRNA is mainly restricted to activated mature B cells [31], thus limiting its function to the cell type and time window where it is required. Transcriptional regulation exerted by B cell specific transcription factors and *cis* elements (reviewed in [32], as well as microRNA-mediated post-transcriptional regulation [33], [34], [35] contribute to this expression pattern.

We hypothesized that limiting physiological AID expression levels could provide an additional mechanism to restrict its deleterious activity. To address this question we analysed the impact of AID reduction in AID^+/−^ mice on CSR, SHM and the generation of chromosome translocations.

## Results

### AID expression is limiting for its function in CSR

**Figure 1:**
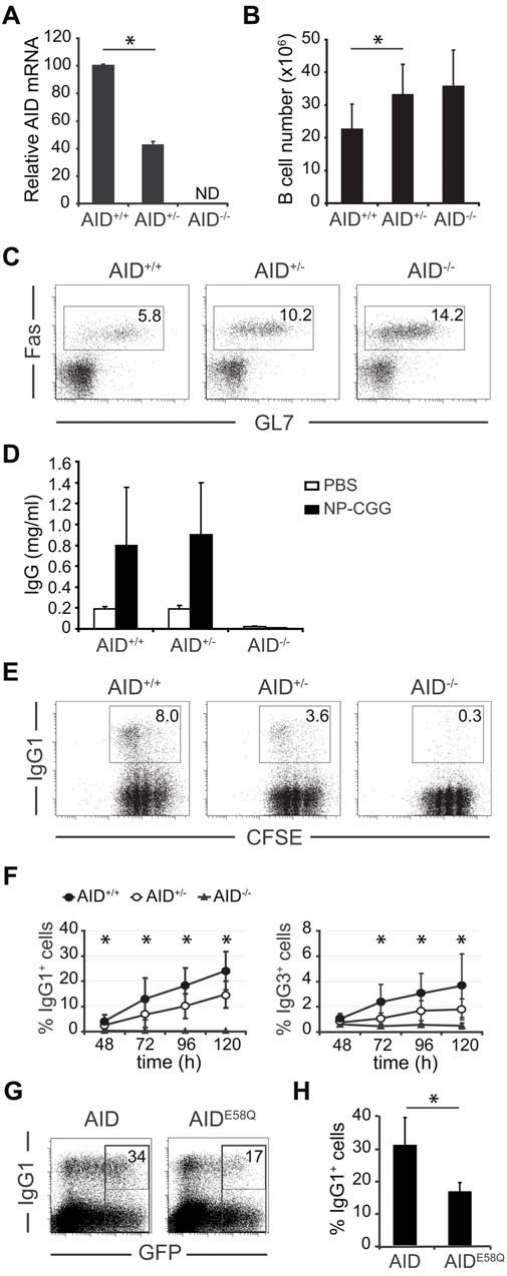
AID is haploinsufficient for CSR. (A) AID levels are reduced in AID^+/−^ mice. Spleen B cells from AID^+/+^, AID^+/−^ and AID^−/−^ were stimulated for 3 days in the presence of LPS and IL4. RNA was isolated and AID expression was assessed by real-time RT-PCR. Bars represent AID mRNA expression relative to AID^+/+^ B cells. Statistical error bars show standard deviation (n = 5). ND, non-detectable. (B) AID^+/−^ spleens contain increased numbers of B cells. Bars represent the number of spleen B cells (CD43 negative) and statistical bars represent standard deviations. n (AID^+/+^) = 18, n (AID^+/−^) = 16 and n (AID^−/−^) = 9. *t* test p (AID^+/+^ *vs* AID^+/^) = 6×10^−4^. (C) Peyer's patch B cells from AID^+/−^ mice contain intermediate Fas^+^GL7^+^ cell numbers. Spleen B cells from AID^+/+^, AID^+/−^ and AID^−/−^ were stained with anti-Fas and anti-GL7 antibodies and analysed by flow cytometry. Percentage of Fas^+^GL7^+^ cells is indicated. One representative experiment is shown (n = 3). (D) ELISA quantification of IgG serum levels in AID^+/+^ (n = 5), AID^+/−^ (n = 5) and AID^−/−^ (n = 3) mice 15 days after NP-CGG (black bars) or PBS (white bars) injection. Statistical bars represent standard deviations. (E) CSR efficiency is reduced in AID^+/−^ B cells. Spleen B cells from AID^+/+^, AID^+/−^ and AID^−/−^ were stimulated for 3 days in the presence of LPS and IL4. CSR to IgG1 was measured by flow cytometry. Representative plots of CFSE labelling and IgG1 expression are shown. Percentage of IgG1^+^ cells is indicated. (F) Time-course analysis of CSR. B cells from AID^+/+^, AID^+/−^ and AID^−/−^ were stimulated for the indicated times (X axis) in the presence of LPS and IL4. Percentage of IgG1^+^ as measured by flow cytometry is represented (Y axis). Statistical bars show standard deviations. p values (AID^+/−^ vs AID^+/+^) for IgG1: 48h, 0.04; 72h, 0.03; 96h, 6×10^−3^; 120h, 3×10^−3^; for IgG3: 48h, 0.1; 72h, 8×10^−3^; 96h, 0.02; 120h, 0.02 (unpaired two-tailed Student's t test, n = 9). (G–H) AID overexpression increases CSR. Spleen B cells from AID^+/+^ mice were transduced with retroviral vectors encoding wild type (AID) or a catalitically inactive (AID^E58Q^) AID along with GFP to monitor transduction. CSR to IgG1 was measured by flow cytometry 2 days after transduction. (G) A representative flow cytometry plot is shown in panel G. Percentages of IgG1^+^ cells within the GFP^+^ population are shown. (H) Percentage of IgG1^+^ cells within AID or AID^E58Q^ GFP^+^ cells determined in 4 independent experiments. Statistical bars represent standard deviations. Statistically significant differences (p< = 0.05) are indicated with a (*) (A–H).

In order to assess whether physiologic AID expression level is limiting for its function, we evaluated AID gene dose effect in mice harbouring one or two functional alleles of the AID gene, AID^+/−^ and AID^+/+^ mice, respectively. B cells from congenic Balb/c ByJ animals [21] were used to minimize strain-to-strain variations. We first wanted to ascertain that deletion of one AID allele in AID^+/−^ mice resulted in a reduction of AID levels as compared to AID^+/+^ mice. Mature B cells were isolated from spleens from AID^+/+^ and AID^+/−^ mice, stimulated *in vitro* in the presence of LPS and IL4 to promote AID transcriptional activation, and AID expression was quantified after 3 days of culture by real-time RT-PCR. We found that indeed AID mRNA levels are reduced roughly to 50% in AID^+/−^ as compared to wild type AID^+/+^ B cells (Figure 1a). AID deficient mice display a mild B cell hyperplasia and enlarged germinal centers [6]. We found that AID^+/−^ mice have slightly increased numbers of B cells in spleen as compared to AID^+/+^ (Figure 1b) and contain a higher proportion of germinal center B cells in Peyer's patches, as measured by the expression of the GL7^+^Fas^+^ activation markers (Figure 1c).

The above results indicate that AID^+/−^ cells express reduced levels of AID mRNA and show hyperactivation features that are intermediate between AID proficient (AID^+/+^) and AID deficient (AID^−/−^) cells.

To address if reduced AID expression in AID^+/−^ B cells results in a decrease of serum Ig levels, we immunized AID^+/+^, AID^+/−^ and AID^−/−^ mice with NP-CGG and analysed Ig concentration by ELISA. In agreement with previously reported data [6], we found no significant difference in serum Ig concentration between heterozygous and fully deficient AID mice (Figure 1d), presumably due to the high variablity in Ig titers. This result is consistent with the finding by Takizawa et al that decreased Ig titers in immunized AID^+/−^ mice is only detected for antigen-specific high affinity antibodies [36]. To directly measure the impact of AID reduction on the induction of CSR, B cells isolated from AID^+/+^, AID^+/−^ and AID^−/−^ spleens were stimulated in the presence of LPS and IL4 to promote CSR to IgG1. FACS analysis after 2 days of stimulation showed that AID^+/−^ B cells show a reduction in the efficiency of CSR to IgG1 (Figure 1e) which parallels the observed reduction of AID mRNA levels (Figure 1a). CSR reduction in AID^+/−^ compared to AID^+/+^ B cells can be observed throughout the duration of the culture (Figure 1f) both for IgG1 isotype (LPS+IL4 stimulation, graph on the left) and for IgG3 isotype (LPS stimulation, graph on the right), and was not associated with proliferation defects (Figure 1e and not shown).

We next asked if surpassing the level of physiologic AID expression would conversely result in an increase of CSR. To approach this issue we transduced spleen B cells from wild type mice with retroviruses encoding AID or AID^E58Q^ catalytically inactive mutant, together with GFP for tracking purposes. We found that AID, but not AID^E58Q^ overexpression, promotes an increase in the efficiency of CSR, as measured by the expression of IgG1 after stimulation in the presence of LPS and IL4 (Figure 1g and h).

From these results we conclude that AID gene dose affects the efficiency of CSR in primary B cells, which implies that the AID gene is haploinsufficient for CSR.

### AID expression is limiting for SHM

**Figure 2:**
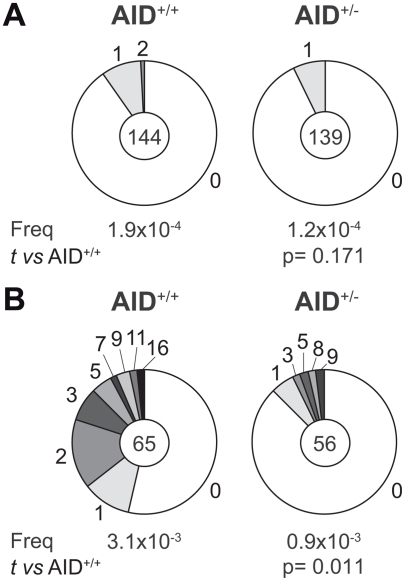
AID is haploinsufficient for SHM. (A) Sμ mutation frequency in AID^+/+^ and AID^+/−^ activated B cells. The Sμ mutation frequency was quantified in sorted CFSE-labelled spleen B cells that had undergone 5 or more divisions after 96h of LPS and IL4 stimulation. Segment sizes in the pie charts are proportional to the number of Sμ sequences carrying the number of mutations indicated in the periphery of the charts. The total number of independent sequences analyzed is indicated in the center of each chart. The calculated mutation frequency per base pair is indicated underneath. Statistical significance was determined by a two-tailed Student's *t* test comparing the frequency found in AID^+/+^ and AID^+/−^ B cells. P values are indicated. (B) SHM analyzed in the intronic region 3′ of JH4 of germinal center B cells from AID^+/+^ and AID^+/−^ mice. Segment sizes in the pie charts are proportional to the number of mutations found in the intronic region 3′ of JH4 of Fas^+^GL7^+^ sorted B cells from Peyer's patches of aged-matched AID^+/+^ and AID^+/−^ mice. The total number of independent sequences analyzed is indicated in the center of each chart. The calculated mutation frequency per base pair is indicated underneath. Statistical significance was determined by a two-tailed Student's *t* test comparing the frequency of mutations found in AID^+/+^ and AID^+/−^ mice. P values are indicated.

To assess whether AID haploinsufficiency was also evident on its activity in SHM we first analysed the accumulation of mutations in the 5′ end of the μ switch region (Sμ). AID activity during CSR leads to the introduction of mutations in Sμ. We stimulated CFSE-labelled spleen B cells from AID^+/+^ and AID^+/−^ mice in the presence of LPS and IL4 for 96h and quantified the accumulation of mutations in cells that had undergone 5 or more divisions after sorting, DNA extraction, PCR amplification, cloning and sequencing. We found a lower mutation frequency in the Sμ region of AID^+/−^ when compared to AID^+/+^ cells (1.2×10^−4^ *vs* 1.9×10^−4^) although this difference was not statistically significant (*t* test p = 0.171) (Figure 2a). We next examined the SHM frequency *in vivo* by isolating Fas^+^GL7^+^ germinal centre cells from AID^+/−^ and AID^+/+^ peyer's patches and sequencing of the intronic region immediately downstream of the JH4 gene. Our analysis showed that B cells from AID^+/−^ mice contain fewer mutations than AID^+/+^ cells (0.9×10^−3^ *vs* 3.1×10^−3^ *t* test p = 0.011) (Figure 2b). From these results we conclude that AID is also haploinsufficient for SHM and therefore that the physiologic level of AID expression is limiting for the diversification of antibodies.

### B cells from IL6tgAID^+/−^ hyperplastic lymph nodes harbour fewer translocations than IL6tgAID^+/+^ B cells

IL6 transgenic (IL6tg) mice develop lymphoid hyperplasia, presumably resulting from IL6-induced proliferation protection of mature B cells from apoptosis. Hyperplastic lymphoid tissues from IL6tg mice are enriched in B cells that harbour chromosomal translocations involving the IgH locus and the c-myc proto-oncogene (c-myc/IgH), analogous to those found in human Burkitt lymphoma. In the absence of AID, IL6tg B cells are devoid of c-myc/IgH translocations and the onset of lymphoid hyperplasia is delayed [19], [21]. Breakpoints of c-myc/IgH translocations found in IL6tg mice cluster in a narrow region of the c-myc gene, which encompasses part of its first exon and first intron. In contrast, translocation breakpoints have been found spreading over a large region of the IgH locus, from the V-J_H_ region to the alpha switch (Sα), which precedes the most distal alpha constant (Cα) segment [19], [21], [37]. This distribution likely reflects the sites of AID-initiated double strand breaks during CSR and on occasion, during SHM.

**Figure 3:**
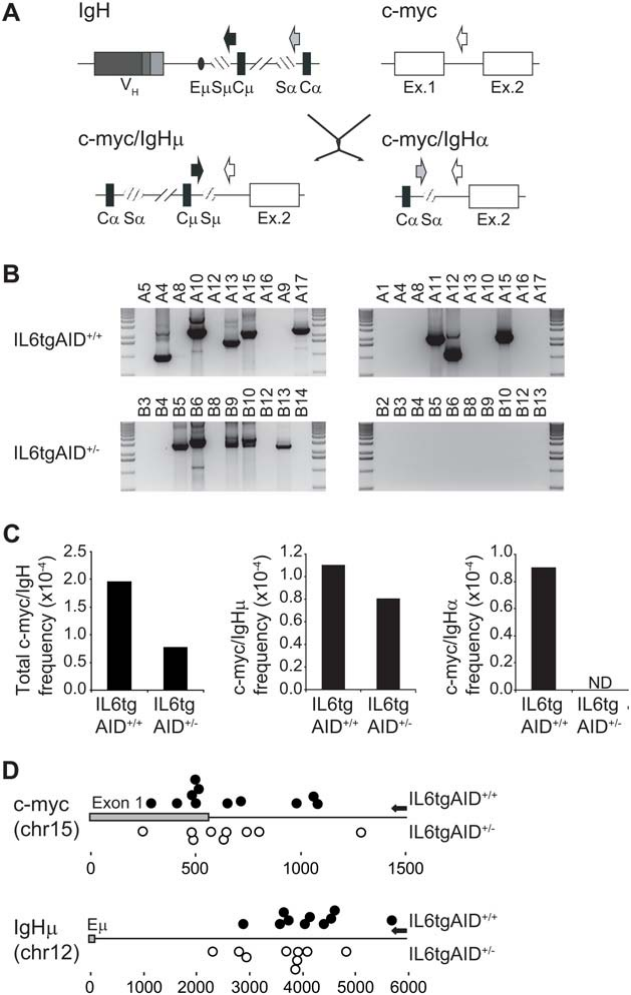
c-myc/IgH translocation frequency is reduced in IL6tgAID^+/−^ mice. (A) Schematic representation of the IgH and c-myc genes (upper) and the derivative chromosomes (c-myc/IgHμ and c-myc/IgHα, lower) arising from proximal and distal translocations, respectively. Variable (V_H_) and constant (Cμ and Cα) genes are represented as grey and black boxes, respectively. Sμ and Sα switch regions and Eμ enhancer are shown as striped and black ellipses, respectively. C-myc exons are drawn as white boxes. Arrows show the position of primers used for PCR amplification. (B) Proximal (left) and distal (right) c-myc/IgH translocations detected in IL6tgAID^+/+^ (upper gels) and IL6tgAID^+/−^ (lower gels) mice. DNA from IL6tgAID^+/+^ and IL6tgAID^+/−^ hyperplastic lymph node B cells was amplified as described in materials and methods using the primers depicted in (A). Representative amplification products analysed in ethidium bromide stained gels are shown. Mouse identifications are shown above the lanes. (C) Frequency of c-myc/IgH translocations in IL6tgAID^+/+^ and IL6tgAID^+/−^ mice. Translocation frequency was determined by serial dilution of DNA samples, followed by PCR amplification, cloning and sequencing. Graphs show the overall translocation frequency (left), frequency of proximal c-myc/IgHμ translocations (middle) and frequency of distal c-myc/IgHα translocations (right). (D) Representation of translocation breakpoints at the c-myc and IgHμ genes found in IL6tgAID^+/+^ and IL6tgAID^+/−^ B cells. Amplification products of proximal c-myc/IgH translocations were cloned and sequenced. Translocation breakpoints at the c-myc (upper diagram) and IgH (lower diagram) genes are shown as closed (IL6tgAID^+/+^) and open (IL6tgAID^+/−^) circles. C-myc exon 1 and IgH Eμ enhancer are represented as grey boxes and distance to these elements is shown underneath (bps). Arrows on the right indicate the position of the PCR oligonucleotides used for amplification.

To determine if AID gene dose has an influence on the frequency or distribution of c-myc/IgH translocations *in vivo* we analysed B cells from IL6tgAID^+/+^ and IL6tgAID^+/−^ hyperplastic lymph nodes by long-range PCR, cloning and sequencing. Oligonucleotides priming at the mu switch (Sμ) region or at the Sα region of the IgH were combined with c-myc oligonucleotides to detect proximal, c-myc/IgHμ or distal, c-myc/IgHα, translocations, respectively [38] (Figure 3a). We found that the total frequency of c-myc/IgH translocations was reduced in IL6tgAID^+/−^ (0,77×10^−4^) B cells when compared to IL6tgAID^+/+^ (1,95×10^−4^) (Figure 3c, left).

We readily detected proximal c-myc/IgHμ translocations in both IL6tgAID^+/+^ and IL6tgAID^+/−^ B cells, in agreement with previous reports [21], [37] (Figure 3b). When the frequency of c-myc/IgHμ translocations was calculated by performing serial dilutions of B cell samples from hyperplasic lymph nodes, we found that it was slightly reduced in IL6tgAID^+/−^ when compared with IL6tgAID^+/+^ cells (Figure 3c, middle; 1.2×10^−4^ *vs* 0.8×10^−4^). Breakpoints from IL6tgAID^+/+^ and IL6tgAID^+/−^ translocations were mapped and characterized by cloning and sequencing (Figure 3d and Table 1). We found that translocation breakpoints at the c-myc gene mostly clustered at the end of the first exon and the beginning of the first intron, regardless of the genotype being analysed (Figure 3d). Breakpoints at the IgH locus were slightly more proximal to Eμ in the case of IL6tgAID^+/+^ B cells (Figure 3d), although this difference was not statistically significant. Both mutation frequency nearby translocation junctions and number of microhomology nucleotides at junctions were similar in IL6tgAID^+/+^ and IL6tgAID^+/−^ B cells.

**Table 1:** c-myc/IgH chromosomal translocations in IL6tg mice

Transloction^a^	ID^b^	Genotype	c-myc^c^	Jnc^d^	IgH^e^
IgHμ	A1	AID^+/+^	AAACAGCTCGAGGAaCTCTTTTCAG	G	GCTGGGGTGAGCTGAGCTGAGCTGG
IgHμ	A2	AID^+/+^	CTTTCCTCTGTCATCTTGACAAGTC		CTGAGCTGGGTGAGCTGAGCTGAAC
IgHμ	A3	AID^+/+^	GCCTTCAAACAGCTCGAGGAGCTCT		CTGGATTGAACTGAGCTGTGTGAgC
IgHμ	A4-1	AID^+/+^	AATCCAGCCTTCAAACAGCTCGAGG	AGC	CCTGCCTGCCTTAAGAGTAGCAACA
IgHμ	A4-2	AID^+/+^	GGACGGGTTGCGATCGCCGCtGGGG		TGAGCTGGGGTGAGCTGAGCTGGGG
IgHμ	A10	AID^+/+^	TTGGGGAGAGTGGGCGGCAGGCTCG		TGGGATGAGGTAGGCTGGGATGAGC
IgHμ	A11	AID^+/+^	AGAGCTGATCGCGGGCAGAGGCAGA	G	GCTGGCCTGGCTGATGAGCTAAGCT
IgHμ	A13	AID^+/+^	AGCCTGGGGAGTCCTGTCCTGGCTC	GC	TGGGGTGAGCTGAGCTGAGCTGAGC
IgHμ	A15	AID^+/+^	TAAAAGGCTCAGGGACGGGTTGCGA		GAGCTGAGCTGGGGTGAGCTGAGCT
IgHμ	A17	AID^+/+^	ACTCTTGAGAAAAGTGTCAAAAaCA	C	TGGGGTGAGCTGAGCTGAGCTGAGC
IgHμ	B2	AID^+/−^	CTAGCAATTCAGGGGCGCGAGGCAT	A	GCTGAGCTGAGCTGAGCTGGGTGAG
IgHμ	B5	AID^+/−^	CTCTTGAGAAAAGTGTCAAAAGtAC	A	aCTGAGCTGAGCTGAGCTGGGGTGA
IgHμ	B6	AID^+/−^	CACTCCAGAGCTGCCTTCTTAGGTC	GC	TGAGCTGGGGTGAGCTGGGCTGAGC
IgHμ	B7	AID^+/−^	AAGAACACAGGGAAAGACCACCAGA		GCTGGGGTGAGCTGAGCTGAGCTGG
IgHμ	B9	AID^+/−^	TCAAAATGCATCCCGGTTTTTCCCT	T	GGGCTGAGCTGGGTTGGGAGACCAT
IgHμ	B10	AID^+/−^	CTCTTTTCAGGAGAGCTGATCGCGG	GC	TGGGGTGAGgTGaGCTGAGCTGGGG
IgHμ	B11	AID^+/−^	AAAGCACAGGAATGGAAGAAAGACT	G	CTGAGCTGGGGTGAGCTGAGCTGAG
IgHμ	B13	AID^+/−^	CAAGAATGTCCAACCGGCCGGGTCA	G	AGCTGAGCTGGGGTGAGCTGAGCTG
IgHα	A11	AID^+/+^	ATCTTGACAAGTCGCTCTACCCCGA	CT	GAGCTAGACTTANGGTGNAATGGGC
IgHα	A12	AID^+/+^	GGGATTGGCGGGCGGGACGCACCTC	C	TAGgCTGGGGTGAATTAGCATGACT
IgHα	A15	AID^+/+^	AACCGGCCGCTACATTCNAGACGCA	G	CTGGGCTGAGCTGGAATGAGCTGGG

a Proximal (IgHμ) or distal (IgHα) c-myc/IgH chromosomal translocation.

b Mouse identification as referred to in figure 2b.

c c-myc sequence adjacent to translocation junction. Mutations are shown in lowercase.

d Microhomology at junctions (overlapping sequences that cannot be assigned to a single sequence, c-myc, or Ig).

e IgH Reverse complementary sequence adjacent to translocation junction. Mutations are shown in lowercase.

In contrast to c-myc/IgHμ proximal translocations, we found that distal c-myc/IgHα translocations, while present in 20% of the IL6tgAID^+/+^ samples, were not detected in any of the IL6tgAID^+/−^ mice analysed (Figure 3b–c, right). We conclude that *in vivo*, the reduction of AID expression in IL6tgAID^+/−^ mice results in a shifted pattern and reduced frequency of c-myc-IgH translocations.

### The frequency of c-myc/IgH translocations generated *in vitro* is reduced in AID^+/−^ B cells

Lymphoid hyperplasia generated in IL6tg mice is a long latency disease presumably involving complex selective events which can bias the number and nature of the translocations found in sick animals. In order to assess more faithfully the effect of limiting AID gene dose on the generation of chromosomal translocations, we analysed the frequency of these events as generated after short *in vitro* cultures of primary B cells. C-myc/IgH translocations are initiated in B cells through the same molecular pathway as CSR, which involves cytosine deamination by AID, and UNG. This process can be recapitulated *in vitro* by stimulating spleen B cells in the presence of LPS and IL4. The frequency of these events in wild type B cells is extremely low (below one translocation every ten million cells), but it is increased in the absence of p53, p19^ARF^ or ATM, reflecting that DNA damage and oncogenic stress pathways are activated downstream of AID function to prevent aberrant joinings or spreading of cells that harbour lymphomagenic translocations. In particular, p53-mediated protection against AID-triggered c-myc/IgH translocations seems to require both alleles of the tumor suppressor, since p53^+/−^ B cells display a dramatic increase in translocation frequency when compared to wild type littermates. Therefore we decided to exploit the higher frequency of c-myc/IgH translocations found in p53^+/−^ B cells to determine whether restricting AID expression levels has an impact on the occurrence of these events.

**Figure 4:**
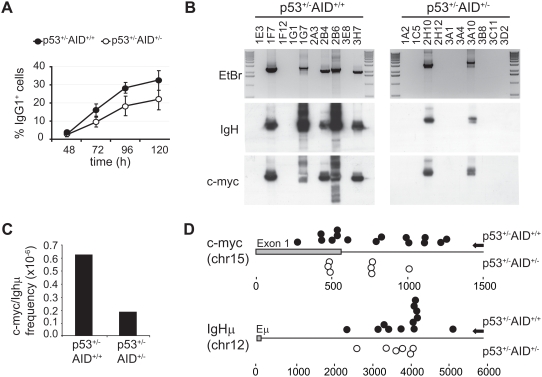
c-myc/IgH translocation frequency is reduced in activated AID^+/−^ B cells. (A) p53^+/−^AID^+/−^ cells are haploinsufficient for CSR. Spleen B cells from p53^+/−^AID^+/+^ and p53^+/−^AID^+/−^ were stimulated for the indicated times (X axis) in the presence of LPS and IL4. Percentage of IgG1^+^ as measured by flow cytometry is represented (Y axis). Statistical bars show standard deviations. p values (p53^+/−^AID^+/−^ *vs* p53^+/−^AID^+/+^) 48h, 0.7; 72h, 0.01; 96h, 6×10^−3^; 120h, 0.03. (unpaired two-tailed *t* test, n = 4). (B) Representative c-myc/IgH translocations detected in p53^+/−^AID^+/+^ and p53^+/−^AID^+/−^ B cells. Spleen B cells from p53^+/−^AID^+/+^ and p53^+/−^AID^+/−^ mice were stimulated in vitro in the presence of LPS and IL4 for 3 days. DNA was extracted and proximal translocations were amplified by PCR as depicted in Fig 2a (see materials and methods) and analysed in ethidium bromide stained agarose gels (upper). Specificity of amplification products was determined by southern blot and hybridization with IgH (middle) and myc (lower) probes. Results for p53^+/−^AID^+/+^ and p53^+/−^AID^+/−^ cells are shown on the left and right gels, respectively. Translocation identifications are indicated above lanes. (C) Frequency of c-myc/IgH translocations in p53^+/−^AID^+/+^ and p53^+/−^AID^+/−^ B cells. Translocation frequency was determined by serial dilution of DNA samples, followed by PCR amplification, cloning and sequencing. (D) Representation of translocation breakpoints at the c-myc and IgHμ genes found in p53^+/−^AID^+/+^ and p53^+/−^AID^+/−^ B cells. Amplification products of c-myc/IgH translocations were cloned and sequenced. Translocation breakpoints at the c-myc (upper diagram) and IgH (lower diagram) genes are shown as closed (p53^+/−^AID^+/+^) and open (p53^+/−^AID^+/−^) circles. C-myc exon 1 and IgH Eμ enhancer are represented as grey boxes and distance to these elements is shown underneath (bps). Arrows on the right indicate the position of the PCR oligonucleotides used for amplification.

To verify that the p53^+/−^ genotype or the mixed strain of these mice do not interfere with AID haploinsufficiency (described above), we generated p53^+/−^AID^+/+^ and p53^+/−^AID^+/−^ animals and analysed the efficiency of CSR in LPS+IL4 B cell cultures. Expectedly, we found that p53^+/−^AID^+/−^ B cells show a decreased level of CSR when compared to p53^+/−^AID^+/+^ cells, as measured by the expression of IgG1 at different culture time-points (Figure 4a). This reduction is comparable to that observed in Balb/c p53^+/+^ mice (see above).

Translocation frequency was then assessed in p53^+/−^AID^+/+^ and p53^+/−^AID^+/−^ spleen B cells upon LPS+IL4 activation. Spleen B cells were isolated from 10 independent mice (5 mice per genotype), and the presence of translocations was analysed by PCR (depicted in Figure 3a, left) in 90 million cells (45 million cells per genotype) after 3 days of stimulation. Translocation identity was verified after gel analysis, southern blot with IgH- and myc-specific probes (see Figure 4b for representative gels), cloning, and sequencing. We found that the frequency of c-myc/IgH translocations is significantly reduced in p53^+/−^AID^+/−^ B cells as compared to p53^+/−^AID^+/+^ (0,19×10^−6^ *vs* 0,61×10^−6^, *Fisher* test p = 0,0025). We did not find significant differences regarding translocation breakpoints (mapped in Figure 4d), mutations or microhomology at junctions (Table 2) in p53^+/−^AID^+/−^ *vs* p53^+/−^ AID^+/+^ translocations.

**Table 2:** c-myc/IgH chromosomal translocations generated *in vitro*

Transloction^a^	ID^b^	Genotype	c-myc^c^	Jnc^d^	IgH^e^
IgHμ	1F7	AID^+/+^	AGACTGTCCTAACCGGCCGCTACAT	T	GAGCTGAGCTGGGGTGAGCTGAGCT
IgHμ	1G5	AID^+/+^	AGACAAAAATTCCCTCCCCGGAGCC	TG	AGCTGGGTGAGCTGAGCTGGGGTGA
IgHμ	2G7	AID^+/+^	CCGCGGATCCCAAGTAGGAATGTaA	GG	GGTGAGCTGGGGTGAGCTGAGCTGA
IgHμ	6H5	AID^+/+^	AAATTTTACTACGATCACTATTTTT		TGAGCTGAGCTGGGGTGAGCTGAGC
IgHμ	6H8	AID^+/+^	GTCGGTCCCAGGCTGTCAGAAATGC		TGAACTGAATGAGTTTCACCAGGCC
IgHμ	8B6	AID^+/+^	AATGTCCAACCGACCGGGTCAGCGT		GTGAGCTGAGCTGAGCTAGGGTGAG
IgHμ	10G5	AID^+/+^	ACCGGGGTTTCCAACGCCCAAAGGA		G**C**TGAGCTGGGCTGAGCTGGGGTGA
IgHμ	10G12	AID^+/+^	ACAGCTCGAGGAGCTCTTTTCAGct	GAGCTGA	GCTGAGCTGAGCTGAGCTGAGCTGG
IgHμ	10H5	AID^+/+^	GGTTTCCCCCAAGTCAACGAATCGG	T	GAGCTGaGcTGgGgTGAGCTGAGCT
IgHμ	11F4	AID^+/+^	AACAGCTCGAGGAGCTCTTTTCAGc		TGAGCTGAGCtGAGCTGAGCTGAGC
IgHμ	11H7	AID^+/+^	GAAATGCACCAAGCTGAAATTTAAA		GGGGTGAGCTGAGCTAGGGTGAACT
IgHμ	3C9	AID^+/−^	GTCGGTCCCAGGCTGTCAGAAA**C**GC		TGAGCTTgGCTGAGCTAGgGTGAGC
IgHμ	7D3	AID^+/−^	AGTAACCTCGGGAACCCCGCTTCAA		TGAGCTGgGgTGAGCTGaGCTGAGC
IgHμ	8G5	AID^+/−^	TCTTTTCAGGAGAGCTGATCGCGGG		TGAGCTGAGCTGAGCTGGGGTGAGC
IgHμ	8H10	AID^+/−^	AGCTCTTTTCAGGAGAGCTGATCGC	GGG	TGAGCTGAGCTGAGCTGGGGTGAGC
IgHμ	9A4	AID^+/−^	AGTAACCTCGGGAACCCCGCTTCAA		TGAGCTGGGGTGAGCTGAGCTGAGC
IgHμ	9C3	AID^+/−^	TGAGAAAAGTGTCAAAAGCACAaGA	A	GCTGAGCTGAGCTGGGGTGAGCTGg
IgHμ	10A10	AID^+/−^	GAGAGCTGATCGCGGGCAGAGGCAG		GAGCTGGAG-TGAGCTGAGCTGGG*C*T

a Proximal IgHμ c-myc/IgH chromosomal translocation.

b Mouse identification as referred to in figure 2b.

c c-myc sequence adjacent to translocation junction. Mutations are shown in lowercase.

d Microhomology at junctions (overlapping sequences that cannot be assigned to a single sequence, c-myc, or Ig).

e IgH Reverse complementary sequence adjacent to translocation junction. Mutations are shown in lowercase.

From this result we conclude that the reduced AID levels expressed by mice that harbour a single copy of the gene (AID^+/−^) results in a decreased frequency of c-myc/IgH translocations. Therefore, AID is haploinsufficient for the generation of these lymphomagenic lesions.

## Discussion

The progress of the humoral immune response relies on the reshaping of the antibody repertoire upon infection by the introduction of somatic changes into the DNA of Ig genes. Higher affinity antibodies are produced by SHM and new effector capabilities are generated by CSR. SHM and CSR are initiated by AID through the deamination of cytosine residues on antibody genes [6]. Accordingly, AID is a critical enzymatic activity for the development of the adaptive immune response [6], [7]. Recent reports have stated that AID-mediated deamination on DNA can also lead to the generation of unwanted lesions, namely, mutations outside the Ig genes [27], [28], or DNA breaks and chromosomal translocations [19], [20], [21], [22], whose contribution to lymphoid neoplasia has been demonstrated in several *in vivo* models [19], [21], [22], [23], [24], [25]. Therefore AID function is expected to be tightly regulated to prevent the generation of DNA lesions.

Here, we have addressed the question of whether restriction of AID expression levels *in vivo* could play a regulatory role in its activity. We performed our analyses in mice that harbour a single functional allele of the AID gene, which results in a reduction of roughly 50% of AID mRNA levels. This reduction is likely due to a per-cell decrease of AID levels, as it has been shown that AID expression is biallelic [36].

Both in mice and humans, the absence of AID results in a hyper IgM immunodeficiency syndrome that is characterized by the absence of somatic mutations and of Ig isotypes other than IgM. This phenotype is accompanied by lymphoid hyperplasia and enlarged germinal centers. Although the significance of these latter features to the immunodeficiency is unclear, we found that reduction of AID expression in AID^+/−^ mice brings about a mild increase of B cell numbers in the spleen and of germinal center cells in Peyer's patches. More importantly, AID gene dose is indeed limiting for the generation of switched isotypes, as B cells from AID^+/−^ mice have an impaired ability to perform CSR *in vitro*, in agreement with very recently published results [36], [39] This observation is reinforced by the finding that exogenous AID expression results in an increase of the CSR rate. In addition, our results show that AID expression is also limiting for SHM. Altogether these data indicate that AID is haploinsufficient for antibody diversification. In humans, AID deficiency (HIGM2) is considered an autosomal recessive disease [7], [40], [41], [42]. This apparent discrepancy with our data can be explained by the fact that HIGM2 patients have relatively mild and variable clinical phenotypes, possibly due to the contribution of genetic and environmental factors [7], [41], [43]. Among those, it is important to note that numerous different mutations in the AID gene have been identified in HIGM2 patients, whose impact on the efficiency of the CSR and SHM reactions is highly variable [7], [41], [43], [44]. In addition, the number of HIGM2 patients characterised so far is small [45], which makes it very difficult to establish genotype-phenotype relationships. Therefore, it is expected that heterozygous individuals display mild to absence of clinical manifestations.

Our analysis of AID-induced chromosomal translocations shows that elimination of one AID allele reduces the frequency of these aberrations. Of note, c-myc/IgH translocations involving the distal Sα switch region were not detected in B cells from IL6tgAID^+/−^ hyperplasic lymph nodes. Although the sample size does not allow concluding that this absence is absolute, our data indicate that Sα translocations are very infrequent when the amount of AID is limiting. This observation is in agreement with the finding that the frequency of AID mutations in Sα is much lower than in Sμ [46]. In turn, this would imply that, in the presence of a single AID allele, the lesions that trigger a DSB in this region are very rare and fall below our detection limit. Analysis of AID-promoted c-myc/IgH translocations in *in vitro* stimulated B cells has allowed an accurate measurement of the frequency of these events in AID^+/−^ B cells, as opposed to the IL6tg (this study) or pristane induced plasmacytomas in Bcl-xL mice [36], where the contribution of *in vivo* selective mechanisms can not be discriminated. Our results show unequivocally that reduction of AID levels results in a significant decrease in the frequency of these events. This reduction in c-myc/IgH translocations found in AID^+/−^ mice is not accompanied by a shift in the location or nature of translocation breakpoints, which indicates that diminishing AID levels affects only the frequency of targeting, rather than its specificity.

Conversely, we had previously observed that AID overexpression in primary B cells produces a major increase in the frequency of c-myc/IgH translocations. In particular, a ten fold protein increase gave rise to a thousand fold increase in translocation frequency [20]. This observation suggests that surpassing a certain level of AID expression is likely to overwhelm the cellular surveillance pathways that protect against these lesions and to result in their accumulation in a non-linear fashion. Together, these results show that AID haploinsufficiency is also revealed in its deleterious activity as promoter of illegitimate chromosome fusions. Interestingly, previous results have shown a longer median survival for IL6tgAID^+/−^ than for IL6tgAID^+/+^mice (7.9 *vs* 5.5 months) in two independent studies which showed similar survival curves for IL6tgAID^−/−^ mice [19], [21]. Very recently it has been reported that in a Bcl-xL transgenic background, AID^+/−^ mice have a delayed plasmacytoma development in response to pristane injection [36]. Together, these observations indicate that AID haploinsufficiency can be relevant for tumor progression *in vivo*.

In summary, we have found that a half reduction of AID expression in mice harbouring a single AID allele, results in a reduction of AID activity, both in the efficiency of antibody diversification and in the frequency of generation of chromosome translocations. This implies that there is a gene dose effect of AID expression, and therefore, that AID is haploinsufficient. Our findings suggest that restraining the physiologic levels of AID expression can be a mechanism that allows the achievement of an optimal balance between immune proficiency and genome integrity.

## Materials and Methods

### Mice, B cell cultures, flow cytometry and ELISA assays

Congenic Balb/c AID^+/−^ mice were obtained by breeding Balb/c AID^−/−^ mice (Ramiro et al., 2004) with wild type Balb/c mice. p53^+/−^AID^+/−^ mice were obtained by breeding Balb/c AID^−/−^ mice with C57*BL/6*J p53^−/−^ mice (Jackson laboratories). Lymph node samples were obtained from IL6tgAID^+/+^, IL6tgAID^+/−^ and IL6tgAID^−/−^ mice (Ramiro et al., 2004, Dorsett 2007). All experiments with mice were performed following the Animal Bioethics and Comfort Committee protocols approved by the Instituto de Salud Carlos III. B cells were purified from spleens by anti-CD43 immunomagnetic depletion (Miltenyi Biotech), labelled with 5 µm CFSE (Molecular Probes) (when indicated) and cultured in RPMI in the presence of 25 µg/ml LPS (Sigma), 10 ng/ml IL-4 (Peprotech), 10 mM Hepes (Gibco), 50 µM β-mercaptoethanol (Gibco) and 10% fetal bovine serum (Gibco). For class switch recombination and differentiation assays BAFF (20 ng/ml R&D Systems) was also included to the previously described medium. Retroviral transductions were performed in LPS+IL4 stimulated B cells in the presence of 8 µg/ml polybrene. Flow cytometry analysis was performed after staining with anti-GL7-FITC, anti-CD95-PE, anti-CD19-APC, anti-IgG1 biotin and anti-IgG3 antibodies, APC-streptavidin and 7-AAD (all from BD Biosciences) in a FACSCanto flow cytometer (BD Biosciences). P-values were calculated using unpaired Student's t test (GraphPad software). For determination of serum Ig titers, age-matched 7–9 weeks old mice were immunized by footpad injection with 50 µg/ml of NP-CGG (Biosearch Technologies) in complete Freund's adjuvant. Serum was collected from blood samples 15 days after the immunization and IgG titers in AID^+/+^, AID^+/−^ and AID^−/−^ mice were determined using a mouse IgG specific ELISA system (Roche Applied Science).

### Real time quantitative PCR

Total RNA was isolated with Trizol (Invitrogen) after Ficoll gradient centrifugation (Amerham Biosciences) of stimulated B cells and converted into cDNA using random primers (Roche) and SuperScript II Reverse transcriptase (Invitrogen). AID mRNA was quantified using SYBR Green assay (Applied Biosystems). GAPDH amplifications were used as normalization controls. Primers for AID were: 5′-ACCTTCGCAACAAGTCTGGCT-3′and 5′-AGCCTTGCGGTCTTCACAGAA-3′. Primers for GAPDH were: 5′-TGAAGCAGGCATCTGAGGG-3′and 5′-CGAAGGTGGAAGAGTGGGAG-3′. Quantitative PCRs were performed on 7900HT fast real-time PCR system (Applied Biosystems). P-values were calculated using paired Student's t test (GraphPad software).

### Mutation analysis

For analysis of mutations at the Sμ region, CD43^−^ spleen B cells were CFSE labelled, stimulated for 96h in the presence of LPS and IL4 and B cells that had undergone 5 or more cell divisions were isolated by sorting (FACSAria). DNA was extracted and amplified using the oligonucleotides 5′-AATGGATACCTCAGTGGTTTTTAATGGTGGGTTTA-3′ and 5′-GCGGCCCGGCTCATTCCAGTTCATTACAG-3′y for 26 cycles (Pfu Ultra, Stratagene). For JH4 intron mutations, Fas^+^GL7^+^ cells were purified by cell sorting (FACSAria), DNA was isolated and amplified with oligonucleotides 5′ACTATGCTATGGAC-3′ and 5′-CTGGACTTTCGGTTTGGTG-3′ for 9 cycles and then 5′-GGTCAAGGAACCTCAGTCA-3′ and 5′-TCTCTAGACAGCAACTAC-3′ for 21 cycles using Pfu Ultra (Stratagene). Amplification products were purified, cloned and sequenced. Sequence analysis was performed using Lasergene software. P-values were calculated using unpaired Student's t test (GraphPad software).

### Translocations assays

C-myc/IgH translocation detection by PCR was performed as previously described (Ramiro et al., 2004; Kovalchuk et al., 1997) on 1 µg (for p53^+/−^ samples) or 500-4 ng (for IL6tg samples) of genomic DNA. C-myc/IgHμ and c-myc/IgHα translocations were amplified using two rounds of PCR with Expand Long Template PCR system (Roche). Primers for derivative 12 c-myc/IgHμ first round: 5′-TGAGGACCAGAGAGGGATAAAAGAGAA-3′ and 5′-GGGGAGGGGGTGTCAAATAATAAGA-3′; for derivative 12 c-myc/IgHμ second round: 5′-CACCCTGCTATTTCCTTGTTGCTAC-3′ and 5′-GACACCTCCCTTCTACACTCTAAACCG-3′; for derivative 12 c-myc/IgHα first round: 5′-CGTGAATCAGGCAGCCGATTATCAC-3′ and 5′- GGGGAGGGGGTGTCAAATAATAAGA -3′; for derivative 12 c-myc/IgHα second round: 5′-GAGCTGACCAACAGTTCTGGCTGTATAGAC-3′ and 5′-GACACCTCCCTTCTACACTCTAAACCG -3′. PCR products were purified, cloned and sequenced. Translocation sequences were aligned using SeqMan from Lasergene software. PCR products were separated on agarose gels and denatured for 20 min in 0.4M NaOH before transfer to nylon membranes and probed with γ-P^32^-ATP labelled oligonucleotides (IgH probe: 5′-CCTGGTATACAGGACGAAACTGCAGCAG-3′ and c-myc probe: 5′- GCAGCGATTCAGCACTGGGTGCAGG-3′). P-values were calculated using two-tail Fisher's test (GraphPad software).
